# A comparison of clinical- and patient-reported outcomes of the cemented ATTUNE and PFC sigma fixed bearing cruciate sacrificing knee systems in patients who underwent total knee replacement with both prostheses in opposite knees

**DOI:** 10.1186/s13018-018-0757-6

**Published:** 2018-03-15

**Authors:** Brian W. Carey, James Harty

**Affiliations:** 1 Cork University Hospital Group, Wilton, Cork, Ireland; 2 MRCSI FRCSI (Trauma and Orthopaedics) Consultant Orthopaedic Surgeon, Cork University Hospital Group, Cork, Ireland

**Keywords:** Total knee arthroplasty, Patient-reported outcome measures, Range of movement, ATTUNE, PFC

## Abstract

**Background:**

The ATTUNE Knee by DePuy Synthes was introduced in 2013. It is designed to provide better range of motion and address patient-reported instability. The PFC Sigma Knee, an earlier prosthesis by DePuy Synthes, is a common knee replacement with a strong clinical track record. Our aim is to compare the outcomes after primary total knee replacement for end-stage knee osteoarthritis of the PFC and ATTUNE knee systems in 21 patients who each have prosthesis in opposite knees using WOMAC, Oxford Knee and SF-12 scores and evaluation of range of motion.

**Methods:**

A review was carried out on 21 patients who underwent primary total knee replacement with both the ATTUNE and PFC knee systems. These were staged operations performed in the same institution and by the same surgeon. All cases were followed up for a minimum of 6 months. WOMAC, Oxford Knee and SF-12 scores, as well as knee range of motion were recorded preoperatively and at 6 months postoperatively.

**Results:**

There was a significant difference in pre- to 6-month post-operative outcomes in PFC and ATTUNE groups with regard to improvement in range of motion (10° ± 8 and 13° ± 11, respectively). There was also a significant improvement in WOMAC scores (PFC group) and Oxford Knee Scores (ATTUNE group) (8.9 ± 7.7 and 12.1 ± 8.4, respectively). There was a significant improvement in SF-12 Score in both groups (10.1 ± 9.3 for PFC and 15.8 ± 13.3 for ATTUNE). The minimum clinically important difference (MCID) in scoring systems at 6 months was reached by 6 patients in the PFC group and 12 in the ATTUNE group.

**Conclusion:**

A significant difference was demonstrated in clinical outcome at 6 months postoperatively between PFC and ATTUNE knee systems in patients who underwent total knee arthroplasty with both prostheses. Superior results were recorded for the ATTUNE knee system.

## Background

Current demand for total knee replacement is rising every year. The amount of knee replacements annually has surpassed that of total hip replacements [1]. This is a trend that is likely to carry on into the future. TKA is generally a very successful operation with impressive clinical outcomes and survivorship results.

There is a constant race between different healthcare companies to advance the technology employed in prostheses to further improve patient outcomes. Newer implants are regularly introduced, or design features of current implants are adjusted in an effort to achieve this.

The Press fit condylar (PFC) total knee prosthesis (DePuy Synthes) was introduced over 30 years ago. It has received several design updates over the years, but the fixed bearing cemented models represent the gold standard of the class, with impressive patient outcomes and excellent 10-year survivorship. [2] The PFC Sigma is the latest model which boasts “J curve” femoral design—three different tangential radius curves in sagittal profile, single radius curve in coronal profiles and refined femoral box edge and trochlear groove edges.

In 2013, DePuy Synthes launched the ATTUNE Total Knee Replacement System. This new design offered multi-radius transition at distal component to posterior condyle region, a reduced femoral component profile and greater mid flexion stability (http://synthes.vo.llnwd.net/o16/LLNWMB8/US%20Mobile/Synthes%20North%20America/Product%20Support%20Materials/Product%20Information%20Sheets/DSUS-JRC-0514-0188%20Val%20Brief.pdf). It is proposed to offer greater functional benefits and a greater range of movement.

With the many design choices that exist, even from the same manufacturer, orthopaedic surgeons face a selection dilemma when little is known on the safety and functionality of newer designs. [3]

## Methods

Ethical approval was granted from the hospital ethics committee as well the Clinical Research Ethics Committee (CREC), Cork. We undertook a retrospective case series of 21 patients who underwent total knee arthroplasty with both the cemented ATTUNE and PFC Sigma fixed bearing, cruciate sacrificing knee systems. A review of patient medical notes was carried out on 21 patients who underwent primary total knee replacement with both the ATTUNE and PFC knee systems, one in each knee. These were staged operations performed in the same institution and by the same surgeon. All operations were conducted with the patient under spinal anaesthetic using the same technique of medial parapatellar approach and capsulotomy. The operations were carried out between 2012 and 2016. All cases were followed up for a minimum of 6 months.

Patient-reported outcome measures as well as knee range of motion were recorded preoperatively and at 6 months postoperatively. Due to a change in clinical follow-up protocol at SIVUH in 2015, different scoring systems were used in clinical assessment. The WOMAC score was used to assess PFC knees whereas the Oxford Knee Score (OKS) was used for ATTUNE knees. The number of patient who reached the minimal clinically important difference (MCID) in the respective scoring systems was also calculated (smallest change in a treatment outcome that a patient would identify as important). The MCID scores for WOMAC and OKS are 11.5 and 6 points, respectively [4, 5].

Maximum passive knee range of motion (ROM) was measured at pre- and post-operative outpatients’ clinic visits using a goniometer.

Statistical analysis was performed using SPSS 23.0 (IBM) at a level of significance of 0.05. Continuous variables were assessed by independent *t* tests. Descriptive statistics are displayed as means with standard deviation or range for continuous variables.

## Results

Data collection was completed in November 2016. A total of 21 patients (9 male and 12 female) were included. The average patient age at the first and second TKR surgeries were 64.3 and 67.6 years, respectively.

All patients completed pre- and 6-month postoperative patient-reported outcome measure questionnaires. (Results: Tables 1, 2, and 3). The mean pre-operative WOMAC score for patients undergoing PFC TKR was 61.2 (range 57.3–76.2). The mean post-operative WOMAC score for these patients was 52.3 (36.1–53.2). The difference between pre- and post-operative scores was found to be statistically significant, *p* = 0.045.

**Table 1:** Pre- and post-operative outcome measures for the PFC group

Outcome measure	Pre-operative		Post-operative		Difference in means	*p* value
	Mean	Range	Mean	Range		
WOMAC	61.2	57.3–76.2	52.3	36.1–53.2	15.4	0.045
SF-12 PCS	29.1	22.3–30.5	39.2	35.6–40.1	10.1	0.032

Paired *t* test

**Table 2:** Pre- and post-operative outcome measures for the ATTUNE group

Outcome measure	Pre-operative		Post-operative		Difference in means	*p* value
	Mean	Range	Mean	Range		
OKS	29.3	26–33	41.4	34–45	15.4	0.039
SF-12 PCS	28.3	26.2–29.4	44.1	38.2–45.3	15.8	0.036

Paired *t* test

**Table 3:** Pre- and post-operative maximum passive flexion values for both groups

Outcome measure	Pre-operative		Post-operative		Difference in means	*p* value
	Mean	Range	Mean	Range		
PFC ROM	95°	89°°–105°	103°	95°–114°	+ 8°	0.039
ATTUNE ROM	94°	80°°–108°	107°	90°°–118°	+ 13°	0.036

Paired *t* test

The MCID for the WOMAC score between pre- and post-operative scores was reached by six patients.

The mean pre-operative OKS score for patients undergoing ATTUNE TKR was 29.3 (range 26–33). The mean post-operative OKS score for these patients was 41.4 (34–45). The difference between pre- and post-operative scores was found to be statistically significant, *p* = 0.039.

The MCID for the OKS score between pre- and post-operative scores was reached by 12 patients.

Preoperatively, both the ATTUNE and PFC Sigma knees had similar maximum passive flexion values (94°, range 80°–108°, and 95°, range 89°–105°, respectively).

At 6-month follow-up, the maximum passive flexion values for the ATTUNE and PFC knees were 107° (range 90°–118°) and 103° (95°–114°), respectively. The + 8° improvement for the PFC knee and the + 13° improvement for the ATTUNE knee were both found to be statistically significant (*p* value 0.039 and 0.036, respectively).

The SF-12 scores showed a significant improvement in both groups (10.1 ± 9.3 for PFC and 15.8 ± 13.3 for ATTUNE).

## Discussion

Healthcare providers worldwide are placing an increased focus on improving patient satisfaction following total knee arthroplasty. It is paramount that innovations and design modifications in newer implants provide the opportunity to improve outcomes.

The goal of this study was to examine these areas by comparing the ATTUNE with the PFC SIGMA knee system. To our knowledge, this is the first study to compare both the ATTUNE and PFC prostheses in the same patient. Previous studies have shown isolated improvements in range of motion but failed to show a significant difference in patient-reported outcome measures. [3, 6]

A positive outcome following total knee arthroplasty is multifactorial. Limited postoperative function [7] and most commonly residual pain [8] are key determining factors.

Patellofemoral maltracking, tilt, and overstuffing have been shown to be important factors leading to pain post TKR [9]. This highlights the significance of implant design in determining patella kinematics. Factors such as trochlear groove geometry and patellar component shape have been shown to play a major role in outcome. [10–12]

Key elements of the design of the ATTUNE knee system are to enhance motion, function and stability. The prosthesis was designed to provide a higher degree of anatomical conformity regarding the trochlear groove and patella. In comparison to the PFC, a recent study has demonstrated a 3° flatter trochlear groove in the ATTUNE [12]. The anatomic, medialised patella of the ATTUNE contrasts with the dome-shaped patella of the PFC. A higher degree of anatomic concordance may improve patient satisfaction.

Limitations of this current study include short-term follow up and a relatively small sample size. Long-term outcome studies will be needed to focus on results at 1- and 2-year follow up. The study only includes a single surgeon. While this may ensure consistency in surgical technique, etc., data from a larger number of surgeons would be preferable. As the operations in this study were staged, comparison is not done during the same period of time. The PFC Sigma has been in use for a longer period of time compared to the ATTUNE; as a result, there is a disparity in terms of long-term follow up studies between the two devices currently.

## Conclusion

The patient group in this study showed superior outcome from their ATTUNE TKR compared to their PFC TKR. Both patient-reported outcome measures and improvements in range of motion highlighted this with statistically significant results.

We acknowledge that there may be a degree of confirmation bias for PROMs in patients having their second total knee replacement, but the improvement in knee flexion showed objective, superior results for the ATTUNE.

While these initial results are promising, long-term outcome studies will be needed to focus on results at 1 and 2-year follow-up.

## Abbreviations

MCID: Minimum clinically important difference
PFC: Press fit condylar
PROM: Patient-reported outcome measure
ROM: Range of movement
TKA: Total knee arthroplasty
TKR: Total knee replacement
WOMAC: Western Ontario and McMaster Universities Osteoarthritis Index
OKS: Oxford Knee Score
SIVUH: South Infirmary Victoria University Hospital
